# Temporal dynamics of a local fish community are strongly affected by immigration from the surrounding metacommunity

**DOI:** 10.1002/ece3.1369

**Published:** 2014-12-17

**Authors:** Rick J Stoffels, Kenneth Robert Clarke, Danielle S Linklater

**Affiliations:** 1 The Murray-Darling Freshwater Research Centre, CSIRO Land and WaterWodonga, Victoria, Australia; 2 Plymouth Marine Laboratory, The HoePlymouth, PL1 3DH, U.K; 3 The Murray-Darling Freshwater Research Centre, La Trobe UniversityMildura, Victoria, Australia

**Keywords:** Community ecology, flow regime, hydrological connectivity, hydrological fragmentation, migration, neutral theory, patch dynamics, species traits

## Abstract

A 5-year time series of annual censuses was collected from a large floodplain lake to determine how dynamics of the local fish community were affected by changes in hydrological connectivity with the surrounding metacommunity. The lake was disconnected from the metacommunity for 1 year prior to our study and remained disconnected until 3 months before our third annual census, when a flood reconnected the lake to the metacommunity. We determined how changes in connectivity affected temporal dynamics of (1) local community composition and (2) the population composition, condition, and growth of catfish, to shed light on how immigration of other species might affect local population dynamics. Before reconnection, the community was likely shaped by interactions between the local environment and species traits. The reconnection caused significant immigration and change in community composition and correlated with a significant and abrupt decline in catfish condition, growth, and abundance; effects likely due to the immigration of a competitor with a similar trophic niche: carp. The community was slow to return to its preconnection state, which may be due to dispersal traits of the fishes, and a time-lag in the recovery of the local catfish population following transient intensification of species interactions. The dynamics observed were concordant with the species sorting and mass-effects perspectives of metacommunity theory. Floods cause episodic dispersal in floodplain fish metacommunities, and so, flood frequency determines the relative importance of regional and local processes. Local processes may be particularly important to certain species, but these species may need sufficient time between floods for population increase, before the next flood-induced dispersal episode brings competitors and predators that might cause population decline. Accordingly, species coexistence in these metacommunities may be facilitated by spatiotemporal storage effects, which may in turn be regulated by flood frequency.

## Introduction

Metacommunity ecology is the study of how regional (e.g., dispersal) and local (e.g., species interactions) processes interact to drive community dynamics at multiple spatiotemporal scales (Leibold et al. 2004; Holyoak et al. 2005a). Within ecosystems comprised of heterogeneous habitats, two metacommunity perspectives may be particularly relevant (Cottenie 2005); the species sorting and the mass-effects perspectives (sensu Leibold et al. 2004), which essentially lie at opposite ends of a continuum. The species-sorting perspective assumes both dispersal and patch quality affect local community structure, but emphasizes spatial niche separation above and beyond effects of dispersal, and so, assumes dispersal is sufficiently low to allow local communities to match the niches provided in a locality, but not so low that species cannot track changes in local conditions (Leibold et al. 2004; Holyoak et al. 2005b). Under this perspective, local dynamics will primarily be driven by local environmental conditions. The mass-effects perspective also assumes that spatial heterogeneity in patch quality interacts with species' niches to shape local communities, but further assumes dispersal from the surrounding metacommunity has a persistent, significant bearing on local dynamics. Under the mass-effects perspective, immigration rates into local communities can be high enough to allow the local persistence of a population that, in the long term, may experience negative population growth rates. (Mouquet and Loreau 2002, 2003). The mass-effects perspective is an intriguing contribution of metacommunity theory because it shows that when we observe the dynamics of a local community we see not just the effects of the local environment, but the effects of neighboring environments in the region, transported to the local community by dispersal.

However, over the last decade, empirical progress in metacommunity ecology has lagged behind that of theory (Logue et al. 2011). It follows that a great challenge to community ecology is to improve our empirical understanding of how dispersal affects local community processes and dynamics (Chase et al. 2005; Van De Meutter et al. 2007; Logue et al. 2011). There are various approaches empiricists might employ to tackle this challenge, but none of them are without trade-offs. Of the descriptive studies carried out so far, most aimed to describe the spatial structure of a metacommunity and used redundancy analysis to partition the variance into environmental and spatial components (e.g., Cottenie 2005; Beisner et al. 2006; Van De Meutter et al. 2007; Pandit et al. 2009; De Bie et al. 2012). The environmental portion of the variance is that which is correlated with environmental heterogeneity among localities, while the spatial portion is that which is better explained by distances between localities. Accordingly, these spatially extensive studies aim to partition metacommunity structure into components that may be due to (1) effects of the niche dimensions of species (species sorting; environmental) and (2) dispersal of species among localities (mass effects; spatial). Key assumptions of this approach are that one's description of the environment within and among localities captures all variance relevant to species' niches and that any unmeasured environmental variables relevant to species' niches are not correlated with distance between localities (Cottenie 2005). It is unlikely these assumptions are met in many cases, as many studies merely use common physicochemical variables to describe the niche space provided by a locality. Consequently, this approach does not include those dimensions of a species' niche defined by biotic interactions, and so, assumes the niches of species are completely defined by habitat structure. Yet localized species interactions may be important drivers of metacommunity dynamics, even in the absence of environmental heterogeneity (Mouquet and Loreau 2003; Leibold et al. 2004; Chase et al. 2005), a result recently supported by experiments (Verreydt et al. 2012). Due to the large effort allocated to describing spatial structure, these types of studies usually provide a static description of metacommunities; temporal dynamics are rarely described (Ellis et al. 2006), resulting in a poor understanding of the population- and community-dynamic consequences of dispersal (Logue et al. 2011). Indeed, in descriptive studies of metacommunities, dispersal of any species is rarely observed directly, but is instead assumed to be some function of spatial autocorrelation (e.g., Beisner et al. 2006) or species traits (e.g., Urban 2004). This may have contributed to a poor process-based understanding of real metacommunities (Logue et al. 2011). It follows that other approaches are required to complement the growing number of spatially extensive studies; approaches that (1) directly observe actual dispersal events and (2) describe the local population- and/or community-dynamic response to such events.

**Figure 1:**
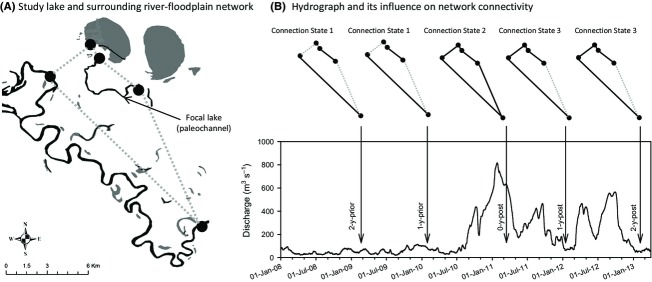
(A) Diagram showing the focal study lake on the floodplain northeast of the Murray River, which flows from southeast to northwest. The superimposed network presents an abstracted view of connectivity among the key waterbodies relevant to this study. Our focal lake (a paleochannel of the Murray River) connects to the Murray River via two creeks (not shown in [A]): via a creek at the northern end of the lake (which connects a large deflation basin lake (in gray) before it connects our study lake); and via a creek at the southern end of the lake. (B) Hydrograph of discharge in the Murray River over the 5-year study, showing how discharge affected connectivity of the river-floodplain network. Diagrams of network connection states indicate the prevailing state prior to each of the five annual sampling periods. Timing of the five study periods is indicated by the arrows. It is clear how the La Niña flood, immediately prior to 0-year-post, affected connectivity of the network.

The fish metacommunities of river-floodplain ecosystems (sensu Junk et al. 1989) are ideal model systems for the investigation of how regional and local processes interact to drive dynamics (Fernandes et al. 2013). A river-floodplain region consists of a high-order river whose adjacent floodplain is dotted with discrete waterbodies of varied habitat quality (Amoros and Bornette 2002). Together, the region can be viewed as a network (sensu Urban and Keitt 2001), whereby the lentic and lotic waterbodies comprise a set of nodes, and hydrological connectivity among those nodes defines the edges of the network (Fig.1). Connectivity of the network is dynamic and is a function of flood height, elevation of the waterbodies, and topography of the terrestrial matrix between waterbodies. Dispersal of fishes throughout the network is episodic, coinciding with overbank flows (Junk et al. 1989). It follows that river-floodplain fish metacommunities may exhibit far-from-equilibrium dynamics, whereby the relative influence of regional and local processes oscillates through time.

Using a five-year time series, the present work describes how a strong dispersal episode affected the local fish community of a large floodplain lake. This analysis was focused on the larger, long-lived species of the community. The consequences of this dispersal event were investigated at both the community and population level. The freshwater catfish, *Tandanus tandanus*, was selected as the focal species for population-level analysis. The objectives were to determine the effect of flood-induced dispersal on local dynamics of (1) community composition; (2) catfish population composition; (3) condition of individuals within the catfish population; (4) mean catfish growth rates. Objectives (2)–(4) were included to improve our understanding of how multispecies immigration might affect the dynamics of a local population, possibly through intensified species interactions. There is a great need to understand the role that species interactions play in biodiversity maintenance. If species interactions are important, then a metacommunity approach to understanding changes in landscape connectivity may be more profitable than a metapopulation approach (Holyoak et al. 2005b).

## Methods

### Site description and fish sampling

Washpen Lake is a large floodplain lake adjacent to the Murray River, Australia (Fig.1A). This is a temperate river-floodplain ecosystem, and so, the flood cycle is irregular and unpredictable (see Bunn et al. 2006). The lake is an historic course (paleochannel) of the Murray River (∼12 km long; ∼40 m wide, on average) and can be reconnected with the Murray during high flows via two natural channels on the floodplain (Taila Creek and Caringay Creek; Fig.1A). Over the 5 years of this investigation, Washpen Lake was in one of three states of lateral hydrological connection (Fig.1B): *Connection State 1*: completely disconnected from the Murray River during low flows; *Connection State 2*: connected to the Murray River via both Taila and Caringay Creeks during very high flows; *Connection State 3*: connected to the Murray River via Taila Creek under moderate flows. This study is concerned with Connection State 2, which was caused by the 2010/11 La Niña flood and lasted for approximately 3 months (hereafter referred to as the La Niña connection).

Fish were sampled at ten sites, each of which was spaced 1.2 km apart and consisted of a 100 m reach of the lake. Both banks of each site were subdivided by ten equidistant sampling points, thus giving 20 potential sampling points along the banks of each site. Fish samples were collected during five “*periods*”: 2 years prior to the La Niña connection (2-year-prior; 2009); 1 year prior to the connection (1-year-prior; 2010); 1 month after the connection (0-year-post; 2011); 1 year after the connection (1-year-post; 2012); 2 years after the connection (2-year-post; 2013). Connection State 1 preceded both 2-year-prior and 1-year-prior (Fig.1B). Connection State 2 occurred immediately prior to 0-year-post (Fig.1B). Following the La Niña connection, Connection State 3 existed until the end of the study and was thus the connection state preceding and during sampling periods 1-year-post and 2-year-post (Fig.1B). We endeavored to ensure each sampling period took place during summers, so that interannual community dynamics were not confounded by season. Unfortunately, the 0-year-post sampling period took place during the autumn, as the flood prohibited earlier access to the system. However, the analysis of Stoffels et al. (2014) showed that seasonal variability in the fish community, although statistically significant, is small relative to the interannual effects of flooding.

Sampling periods consisted of one to three sampling “*events*,” each of which involves the same sampling design (sites, number of samples, etc.), irrespective of which period the event falls in: During each sampling event, 10 single-wing (8 m × 0.65 m) fyke nets (28 mm stretched mesh) were set within each site in the afternoon and retrieved the following morning, with set and retrieve times for each net being recorded such that Catch per Unit Effort (CPUE) could be calculated. The 10 nets were randomly dispersed among the 20 sampling points. Note that temporal community analyses can be confounded by changes in sampling effort, if sampling effort significantly affects the presence–absence structure of the abundance matrix. However, the asymptote of the species accumulation curve indicated that even our minimum sampling effort (100 samples) was sufficient to characterize the presence–absence structure of the community; further sampling merely improved our chances of recapturing tagged catfish (see below).

Sampling period 2-year-prior comprised a single sampling event in February 2009, hence a total of 10 (samples) × 10 (sites) = 100 individual fyke samples. The 1-year-prior sampling period consisted of a single sampling event in December 2009 (100 samples). The three sampling periods following the flood, 0-year-post, 1-year-post, and 2-year-post were comprised of two sampling events, resulting in 200 individual samples for each period. 0-year-post occurred during May 2011 while 1-year- and 2-year-post occurred during January–February 2012 and 2013, respectively. Sites served as replicates for between-period (between-year) comparisons (see Analysis), and the abundance for each site, each sampling period, was mean CPUE calculated across all fykes set at that site within a sampling period (either 10 or 20 nets, as described above).

Upon retrieval of nets fishes were identified to species, enumerated, measured (total and standard length), and weighed before being released. In addition to estimation of length and mass, all catfish ≥ 200 mm TL were implanted with a passive integrated transponder (PIT). These PITs enabled nondestructive estimation of growth rates and the parameters of a von Bertalanffy growth curve for the Washpen catfish population (following Quinn and Deriso 1999).

### Analysis

The approach used here is essentially one of intervention analysis of an unreplicated, large-scale perturbation (Box and Tiao 1975; Carpenter 1990; Eberhardt and Thomas 1991; Stewart-Oaten et al. 1992; Oksanen 2001). In such analyses, hypothesis testing is less important than estimation of effect size and assessment of its ecological importance by the scientist (Hurlbert 1984; Stewart-Oaten et al. 1992; Stewart-Oaten and Bence 2001). Accordingly, the emphasis of our analyses is on effect sizes. Specifically, for both community and catfish population data, we estimate the magnitude of change – the effect size – between the four pairs of contiguous periods in our 5-year time series: (1) 2-year-prior and 1-year-prior; (2) 1-year-prior and 0-year-post; (3) 0-year-post and 1-year-post; (4) 1-year-post and 2-year-post. Although community and/or population structure may change significantly for each of these pairwise comparisons, if the La Niña connection is having a notable impact on the community and population dynamics then we should observe larger effect sizes for comparisons (2) and (3), than for (1) and (4). Note that each site served as a replicate for each of these pairwise comparisons, and so, we had ten replicates for each comparison.

All abundances are in CPUE, which is herein defined in units of number of individuals per net per hour (ind. net^−1^ h^−1^). Nonparametric multivariate analyses were conducted on Bray–Curtis similarities calculated among untransformed samples and were used to test for differences in fish community structure among periods (ANOSIM; Clarke and Green 1988), utilizing the PRIMER v6 package (Clarke and Gorley 2006). We did not transform CPUEs for two reasons: first, mean abundances of the five species were not so dissimilar that the dynamics of one species would dominate whole-community trends; second, changes in abundance as well as composition were of interest. Our ANOSIM model was single factor (five periods), and we were specifically interested in the R-statistics associated with the four pairwise tests mentioned above (the R-value being both a test statistic and an effect size, in an analogous way to a correlation coefficient).

SIMPER (Clarke 1993) and simple shade-plots (Clarke et al. 2014) (utilizing a development version of PRIMER software, v7*α*21, PRIMER-E, Plymouth) enabled us to determine which species had the greatest impact on changes between periods, as well as visualization of replicate variability within a period. Averages over replicates of the CPUE data for the five fish species were then used to compute Bray–Curtis similarities in mean community composition among the five periods, and metric multidimensional scaling (mMDS) used to visualize the community trajectory. Superimposed on the mMDS ordination were segmented bubbles, where segment sizes are proportional to the mean CPUE for each species within a period. Although nonmetric MDS (nMDS) is more commonly used for ordination due to its greater flexibility in representing (dis)similarities on a monotonic scale of distances in the 2-dimensional ordination (Clarke and Warwick 2001), the metric MDS (Cox and Cox 2001) can result in more reliable ordinations when the number of points plotted is low, such as the five period means visualized here. The mMDS and segmented bubble plots were carried out using the test PRIMER v7*α*21 software.

We used a subset of the recapture data to estimate the parameters of a von Bertalanffy growth model for the catfish population (Quinn and Deriso 1999). These parameters were then used to estimate age from lengths, and hence break up the catfish samples from each site and period into five cohorts: 0 + , 1 + , 2 + , 3 + , and 4 + . While the cohort structures for periods 2-year-prior through to 0-year-post were obtained using von Bertalanffy parameters from Stoffels et al. (2014; *n* = 37), those of periods 1-year-post and 2-year-post were estimated using updated parameters, following additional recaptures (*n* = 7) during these last two periods (*n* = 44). The proportions of the total catfish abundance that each cohort comprised at each site, during each period, were then treated as variables in a multivariate analysis. As for the analyses above, we used mMDS for visualization, ANOSIM to determine the magnitude of changes in population structure, then SIMPER and shade-plots to determine which cohorts are contributing most to any change in population structure among sampling periods. Bray–Curtis similarities were calculated on untransformed cohort proportions, and the ANOSIM model was the same as that used for community structure.

We determined the condition of the catfish population for the five sampling periods: Lengths (total length) and masses (g) of all individuals from each period were *ln*-transformed before testing for differences in the intercepts, using analysis of covariance (ANCOVA), where lines were constrained to be parallel (MATLAB Statistics Toolbox R2013a). Significant differences in intercepts indicate significant differences in the length–mass relationships of the samples, hence differences in mean condition of individuals.

We determined mean linear growth rate (g day^−1^) of catfish using recapture data. Define *M*_1*i*_ as the mass of a fish captured at time 1 and *M*_2*i*_ as the mass of the same fish at the time of recapture (time 2). The elapsed time between tagging/capture and recapture of individual *i* is Δ*t*_*i*_ = *t*_2*i*_−*t*_1*i*,_ and let growth of that individual be Δ*M*_*i*_ = *M*_2*i*_−*M*_1*i*_. We calculated the growth rate of individual *i* as 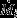 = Δ*M*_*i*_*/*Δ*t*_*i*_. 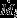 – values were determined only from recaptured fish whose growth was determined over one of two individual years: (1) 2009, the year prior to the flood where *t*_2_ = 1-year-prior and *t*_1_ = 2-year-prior; (2) 2011, the year following the flood where *t*_2 _= 1-year-post and *t*_1 _= 0-year-post. Insufficient catfish were recaptured between 1-year-prior and 0-year-post, and between 1-year-post and 2-year-post to determine growth rates during 2010 and 2012, respectively. Because growth rate is a function of body mass, as well as environmental conditions, we used ANCOVA to compare the regressions between body length and growth rate across years. Regressions were constrained to be parallel and were conducted between ln(length) and ln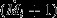 .

## Results

**Figure 2:**
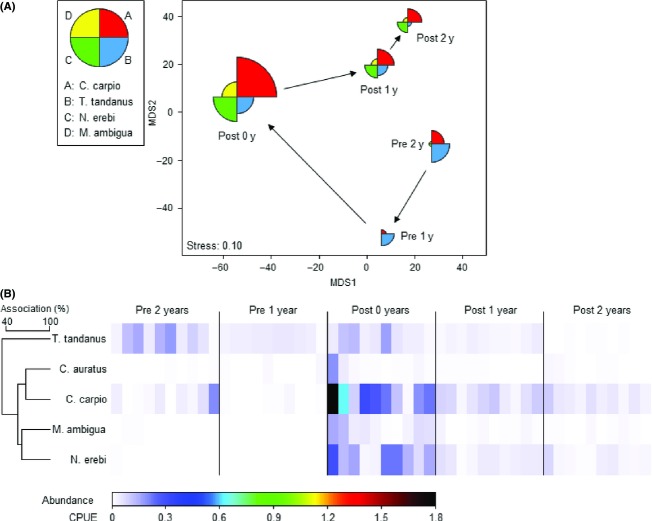
(A) Metric MDS showing trajectory of large-bodied fish community structure in Washpen Creek across 5 years, which were interjected by a large, natural flood; 2 years prior to the flood (Pre-2 years and Pre-1 year), immediately after the flood (Post-0 year), and 2 years after the flood (Post-1 year and Post-2 years). Individual points determined using Bray–Curtis similarity among mean CPUEs of five species (means calculated across ten sites). Points plotted as segmented bubbles, where segment area is proportional to CPUEs (equally scaled). (B) Shade plot for the community matrix plotted in (A): In each row, there are 10 (sites) by 5 (years) = 50 rectangles, shaded according to CPUE at that site, as shown in color key, from white (absent) to black (highest CPUE of 1.8). Rows (species) are ordered according to a cluster analysis based on the Index of Association between species across the 50 samples. Catfish: *Tandanus tandanus*; Goldfish: *Carassius auratus*; Carp: *Cyprinus carpio*; Golden perch: *Macquaria ambigua*; Bony herring: *Nematalosa erebi*.

ANOSIM revealed very strong and significant variation in fish community structure among years (Global *R* = 0.701; *P *<* *0.001; Fig.2). More importantly, pairwise R-values indicated that the changes of greatest magnitude were immediately after the flood and during the year following the flood (“1-year-prior v 0-year-post” and “0-year-post v 1-year-post,” respectively; Table1), a pattern that is particularly clear in the mMDS (Fig.2A).

**Table 1:** *R*-statistics (effect “sizes” or “magnitudes”) of the four key planned pairwise tests associated with the ANOSIMs

Test	Community change	Catfish population change
2-year-prior versus 1-year-prior	0.40**	0.02 ^ns^
1-year-prior versus 0-year-post	0.96***	0.53***
0-year-post versus 1-year-post	0.64***	0.49***
1-year-post versus 2-year-post	0.41**	0.15*

ns, not significant; ^*^P <* *0.05; ^**^P <* *0.01; ^***^P <* *0.001.

SIMPER revealed that increases in the abundance of carp, bony herring, golden perch, and catfish are driving community changes immediately following the flood, together contributing 97% of total dissimilarity between 1-year-prior and 0-year-post. Carp and bony herring alone contribute 50% and 30% (respectively) of the dissimilarity immediately after the flood (Fig.2A and B). Stoffels et al. (2014) provide a fuller description of the community changes observed during 0-year-post. During the year following the flood, declines in the abundance of the same four species were primarily responsible for driving community change, together contributing 97% of total dissimilarity among the 2 years. During the final year (“1-year-post v 2-year-post”), the change in community structure was much more subtle (Table1), but all species continued to decline in abundance (Fig.2A and B).

**Figure 3:**
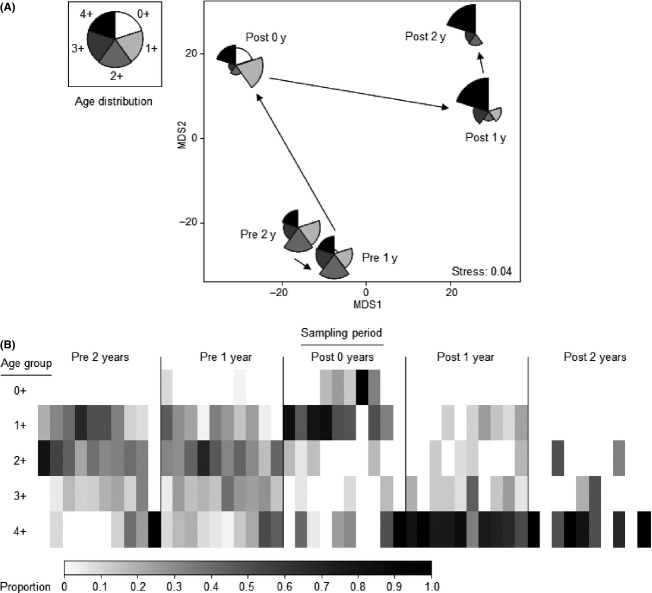
(A) Metric MDS showing trajectory of catfish population structure in Washpen Creek across 5 years, which were truncated by a large, natural flood; 2 years prior to the flood (Pre-2 years and Pre-1 year), immediately after the flood (Post-0 year) and 2 years after the flood (Post-1 year and Post-2 years). Individual points determined using Bray–Curtis similarity among mean proportionate cohort composition (means calculated across ten sites). Points plotted as segmented bubbles, where segment area is proportional to CPUEs (equally scaled) of the five cohorts. (B) Shade plot for the population matrix plotted in (A): In each row, there are 10 (sites) by 5 (years) = 50 rectangles, shaded according to proportion at that site, as shown in the mono shading key, from white (0%) to black (100%).

ANOSIM also revealed strong and significant changes in the composition of the catfish population among years (Global *R* = 0.419; *P *<* *0.001; Fig.3), but more importantly, the pairwise R-values showed that the changes of greatest magnitude were easily those immediately after the flood and during the year following the flood (“1-year-prior v 0-year-post” and “0-year-post v 1-year-post,” respectively; Table1), a pattern that – as observed for the community dynamics – is very clear in the mMDS (Fig.3A). Immediately, after the flood, we observed an increase in the proportion of the population comprised of 0 + , 1 + , and 4 +  individuals, but a decline in 2 +  and 3 +  individuals (Fig.3A and B). During the year, following the flood, there was a strong decline in the proportions of all cohorts except for the 4 +  cohort, which came to dominate the population (Fig.3A and B). This trend continued during the second year after the flood, with the population becoming almost entirely comprised of 4 +  individuals (Fig.3A and B).

**Figure 4:**
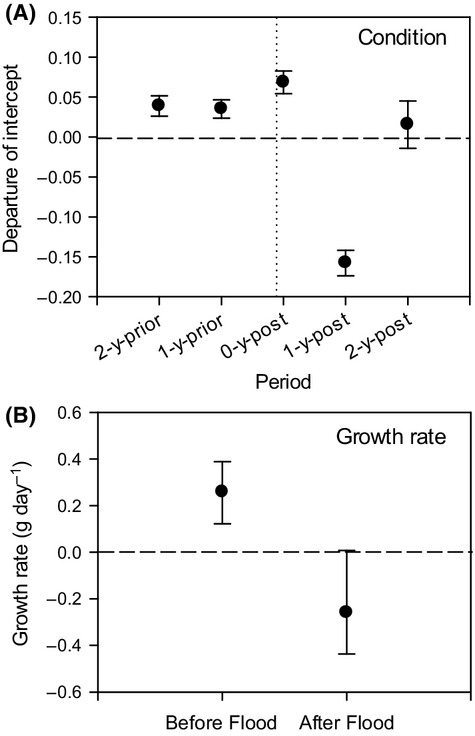
(A) Time series of mean catfish condition across 5 years. Specifically, the magnitude (±SE) and direction of departures of the intercepts of period-specific *ln*(length)-*ln*(mass) regressions, as determined by ANCOVA, assuming equal slopes. Horizontal dashed line indicates zero departure from the global intercept, determined across all periods. Vertical dotted line indicates time of La Niña connection. (B) Mean growth rates (±SE) of catfish before (during 2009) and after (during 2011) the La Niña connection.

The global length–mass regression for catfish – whereby data from all years were treated as a single sample – had a slope (3.19; SE = 0.02) and intercept (−12.77; SE = 0.12) that were significantly different from zero. ANCOVA revealed significant departures of the “year-specific” intercepts from the global intercept of parallel length–mass regressions (*F* = 30.34; *P *<* *0.001; Fig.4A). Most notable was the significantly lower intercept of the 1-year-post catfish, indicating a significant loss of condition from previous years (Fig.4A).

ANCOVA also revealed a significant difference in the intercepts of parallel regressions of *ln*(growth) on *ln*(length) between 2 years, 1 year prior to flooding (2009) and the year immediately after the flood (2011; Fig.4B; *F* = 94.24; *P *<* *0.001). Indeed, while catfish growth was positive in the lake prior to flooding, it became negative during the year after flooding (Fig.4B).

## Discussion

This study shows that community dynamics within floodplain waterbodies may be strongly influenced by episodic immigration from the surrounding river-floodplain network. Even though Washpen Lake is a relatively large floodplain lake, a 3 month connection with the river caused increases in the abundance of carp, golden perch, and bony herring by one to two orders of magnitude. The magnitude of active dispersal required to cause such a community dynamic would have been very large. For example, in a different study (Stoffels, unpublished data), daily migration rates of fish during a 77-day artificial connection between Washpen Lake and the river were estimated, using directional netting in Taila Creek, the only creek to be inundated during that particular event. Modeled immigration rates indicated that the total number of carp that moved into the lake over that period was ∼23,500 individuals (95% CI: 23,000–24,000; unpublished data). Yet that artificial connection had a minor effect on the local community, relative to that of the La Niña connection studied here (Stoffels et al. 2014). One could reasonably conclude, therefore, that dispersal of hundreds of thousands of individuals into the lake must have occurred to drive the community dynamic described here.

Given the magnitude of change in the community trajectory caused by this dispersal event one must ask: how important are local environmental effects, hence species-sorting dynamics, in river-floodplain metacommunities? Numerous researchers have presented evidence that a large proportion of the spatial structure of river-floodplain fish metacommunities may be described by habitat variables (e.g., Rodríguez and Lewis 1997; Winemiller et al. 2000; Sullivan and Watzin 2009). If habitat is the predominant driver of local community structure, then we may expect the local community to return to its preflood state shortly after the dispersal event. This appears to be the case in tropical systems, where the return of local fish communities to preflood states following dispersal pulses is relatively rapid, indicating strong species sorting (Rodríguez and Lewis 1994; Fernandes et al. 2013). Data from Washpen Lake, however, are not concordant with this dynamic. Two years after the La Niña connection, the fish community had not returned to its preflood state. Indeed, the community dynamic presented in Fig.2 appears to indicate random ecological drift (neutrality; Hubbell 2001), whereby the dispersal pulse sends the community trajectory drifting to a new region of community space. Random ecological drift is certainly a valid hypothesis to explain the dynamic presented in Fig.2, but our knowledge of the species traits comprising this community, in addition to the population-level analyses, leads us to present a competing hypothesis, with two major parts: (1) community state prior to the La Niña connection largely reflected matching of species traits with the local environment; (2) the absence of a rapid return to preflood state was due to lingering mass effects of carp, bony herring, and golden perch, coupled with the intensification of local species interactions between catfish and carp, which reduced the abundance of catfish in its favoured local habitat.

### Before the La Niña connection

Prior to the La Niña connection, our study lake had been completely disconnected from the surrounding river-floodplain network for 3 years, and there had been no natural floods for 10 years (Stoffels et al. 2014). The absence of floods for 10 years is significant as floods contain the cues that mobilize pulses of riverine fish dispersal (Schlosser 1998; David and Closs 2002; Nunn et al. 2010; Stoffels et al. 2014). Therefore, dispersal to or from Washpen Lake was impossible during the 3 years prior to the La Niña connection, and dispersal rates were likely very low from 3 to 10 years prior to the connection. It follows that species coexisting within Washpen Lake prior to the La Niña connection must have had traits that interact with the local environment to enable persistence.

Bony herring are a relatively short-lived species (longevity: 2–3 years; Puckridge and Walker 1990; Pusey et al. 2004), and so in the absence of connections to neighboring waterbodies, would have required appropriate spawning habitat within Washpen Lake for local persistence. Bony herring spawn in gently sloping, unvegetated littoral areas of either lotic or lentic waterbodies (Puckridge and Walker 1990; Pusey et al. 2004), but this habitat type is poorly represented in Washpen Lake, which is better characterized by steeper, vegetated littoral areas. Accordingly, while Washpen Lake might represent a profitable foraging habitat for this species in the short term, it is likely a population sink in the long term. The data presented here concur with this explanation, as bony herring were very rare, then undetected, during the 2 years before connection.

Similarly, Washpen Lake does not contain habitat conducive to the spawning of golden perch, which require flowing channels for spawning. Unlike bony herring, however, golden perch generally live longer than 3 years and if local habitat enabled survival, then they could persist in Washpen Lake in the absence of local spawning and recruitment. Golden perch were very rare 2 years prior to the La Niña connection and were not recorded at all 1 year before the La Niña connection. This is concordant with our understanding of the niche of this species, which is generally associated with flowing channels of river-floodplain networks (Pusey et al. 2004), notwithstanding the tendency of juvenile golden perch to disperse onto the floodplain during a flood (see After the La Niña connection).

Carp are a long-lived river-floodplain generalist, and so, in the absence of any spawning and recruitment can persist in most river-floodplain waterbodies for many years (Koehn 2004). Contrasting with bony herring and golden perch, carp do spawn within floodplain waterbodies. However, although this species may exhibit very weak spawning every year, strong spawning events, including associated dispersal behavior, coincide with floods (Brown et al. 2005; Jones and Stuart 2009). The observed local persistence of carp in Washpen Lake prior to the La Niña connection at moderate to low abundances is, therefore, consistent with our understanding of the ecology of the species during prolonged periods of low river-floodplain connectivity.

The decline of catfish following European settlement was so rapid that our understanding of its ecology is poor. Historical anecdotes suggest it may have preferred large floodplain waterbodies and certainly spawned within them (Macleay et al. 1880), and unlike carp, catfish spawning has not been linked to flooding (Davis 1977). Our data concur with this anecdotal and scientific evidence, as it was the dominant species in Washpen Lake prior to the La Niña connection, and spawning and recruitment of catfish were observed in this lake while it was disconnected from the surrounding river-floodplain network. Indeed, Washpen Lake now represents what is a very rare type of waterbody conducive to catfish productivity (large permanent paleochannel lake with a diverse aquatic plant community (Macleay et al. 1880; Stoffels et al. 2014)).

### After the La Niña connection

Dynamics of carp, bony herring, and golden perch abundance following the La Niña connection are concordant with our understanding of their dispersal and life-history traits in response to floods. All three species exhibited a similar temporal dynamic, which could be broadly described as an asymmetric wave, whereby a very large increase in abundance was observed immediately after the flood, but then a more gradual decrease during the following 2 years. Although the temporal changes in abundance were very similar across species, the underlying causes differ: During floods adult carp migrate *en masse* to the floodplain to spawn (Brown et al. 2005; Jones and Stuart 2009). Being a river-floodplain generalist, many individuals return to the river channel after spawning, before lateral hydrological connectivity is lost, while some may remain in floodplain waterbodies. Bony herring and golden perch, on the other hand, appear to be opportunistic floodplain users during floods, whereby numerous individuals – primarily juveniles (Pusey et al. 2004; Stoffels et al. 2014) – rapidly move throughout the river-floodplain network to exploit newly created foraging habitat (Balcombe et al. 2005; Balcombe and Arthington 2009; Puckridge et al. 2010). However, there appears to be a stochastic aspect to their dispersal behavior that results in a substantial portion of individuals being left behind in waterbodies after the dispersal pulse, even if such waterbodies cannot provide suitable habitat for the completion of life-history cycles (spawning, in this case). Because carp spawning is strongly linked to flooding, and the local habitat of Washpen Lake does not support spawning of bony herring and golden perch, we speculate that the local abundance (but not regional abundance) of these three species will continue to decline to some low equilibrium density, as long as floods do not induce another dispersal pulse. Thus, lingering mass effects caused by the dispersal and life-history traits of these three species may be part of the reason why community state 2 years after the La Niña connection had not returned to its preconnection state.

By contrast, dynamics of catfish abundance were surprizing and cannot be explained by dispersal and life-history traits alone, as they are known to be a very sedentary species with strong site fidelity (Reynolds 1983; Pusey et al. 2004). Why, then, did we observe significant changes in the abundance of catfish following the La Niña connection? If local habitat is the primary driver of community structure outside of flood-induced dispersal events, then why did catfish not resume numerical dominance of the local community? We propose that intensified competitive interactions, primarily due to the immigration of carp, may have played a role in driving catfish population dynamics following the connection.

Catfish and carp both have a similar trophic niche (benthic suction feeders; Stoffels 2013), and so, there exists high potential for exploitation and/or interference competition between these two fishes. This fact, coupled with the magnitude of carp immigration during the La Niña connection, may explain the significant and strong decline in mean catfish condition and growth rates during the year following the dispersal pulse. In turn, declining energetic profitability of habitat within Washpen Lake is a likely explanation for the significant and strong decline in catfish abundance during the 2 years following the dispersal pulse of carp, with catfish possibly incurring (1) reduced spawning, (2) reduced recruitment, (3) increased mortality, and/or (4) increased emigration. Our data do not enable us to distinguish between emigration and mortality, but we certainly observed no juvenile (0+) recruitment during the years 1-year-post and 2-year-post La Niña connection, suggesting reduced spawning and/or recruitment may have been at least partly responsible for the decline in catfish abundance.

### A metacommunity view of river-floodplain fishes

Acquiring an empirical understanding of complex metacommunities is a great challenge to ecologists, particularly those working on long-lived vertebrates with active dispersal (Logue et al. 2011). Like any approach one may take to improve our understanding of metacommunity dynamics, the approach taken here has its trade-offs. While this community time series has demonstrated transience of composition in local communities open to regional influences, we were unable to demonstrate how the local dynamics of Washpen Lake relates to dynamics in neighboring localities of the river-floodplain network. Alongside controlled experiments and mathematical models, descriptive studies carried out at large spatial scales make an important contribution to metacommunity ecology. Such studies generate new hypotheses and provide valuable insight into the complex spatiotemporal dynamics that unfold at large scales, but causality is difficult to infer, and generalizations of patterns cannot be made until the study is repeated in different contexts (Hargrove and Pickering 1992; Oksanen 2001). In any case, our data are consistent with a view of river-floodplain metacommunities whereby the rules governing structure alternate through time (concordant with Fernandes et al. 2013). Floods are the key physical process in river-floodplain metacommunities, opening and closing corridors between waterbodies and mobilizing discrete, multispecies dispersal pulses. Accordingly, the structure of local communities may carry a strong regional signal following floods, as dispersal takes the effects of local processes that have accumulated within multiple waterbodies and spreads them throughout the river-floodplain network. As time elapses since floods, species traits likely interact with local (a)biotic environments to shape metacommunity structure. That is, time elapsed since flooding might dictate the relative influence of mass effects and species sorting in shaping river-floodplain metacommunities.

The strong and abrupt change in catfish population structure, including reduced condition and growth, coincident with the immigration of other species, demonstrates that species interactions may play a role in structuring river-floodplain metacommunities. If the hypothesis we presented earlier is true, then flood frequency will regulate the effect of species interactions on the catfish. That is, if Washpen Lake contains habitat conducive to local rates of increase in catfish, then it may be difficult for local habitat to affect catfish population processes if they are regularly being interrupted by flood-induced pulses of immigration of potential competitors. Essentially, we hypothesise that storage effects may be an important mechanism of species coexistence in river-floodplain metacommunities (Chesson 2000; Hoopes et al. 2005). In the context of our study system, local catfish populations may need to “store-up” the benefits of local effects during low-flow periods in order to coexist regionally with other species that have a negative impact on catfish during high-flow periods, whereupon these opportunistic species undertake dispersal pulses.

Clearly, then, a metacommunity view of river-floodplain fishes is pivotal to management of biodiversity within these complex systems. Flood frequency is now under the control of humans in most of the world's river-floodplain systems, which have been regulated for flow control (Opperman et al. 2009). It follows that, under a metacommunity view of biodiversity maintenance, humans are in control of storage effects, hence the coexistence of species and effects of flood frequency need to be further studied in a multispecies context if they are to be managed well. Hydrological connectivity in river-floodplain networks is also under the control of humans in many parts of the world (Opperman et al. 2009). If species interactions play an important role in driving river-floodplain metacommunity dynamics – as suggested here – then understanding the effects of hydrological fragmentation may benefit more from a metacommunity approach than a metapopulation approach (sensu Holyoak et al. 2005b).

In conclusion, the novel time series approach taken here to studying river-floodplain metacommunities was insightful. Dispersal pulses at regional scales may have a strong impact on local community dynamics in river-floodplain metacommunities, and the relative importance of local and regional processes may continually change with the flow regime. By recording time series at two levels of organization, we have shown that multispecies dispersal pulses can affect the age composition of local populations, in addition to the species composition of local communities. In our opinion, metacommunity theory has much to offer both our fundamental understanding and management of biodiversity in river-floodplain systems. However, we contend that progress in our understanding of river-floodplain fish metacommunities will be better facilitated by focusing on how species traits interact with the spatiotemporal structure of the environment at regional and local scales to shape dynamics. In doing so, one would be focusing less on which of the four “perspectives” best applies to a system (patch dynamics; species sorting; mass effects; neutrality; Leibold et al. 2004) and instead testing the various assumptions underlying metacommunity models: How important are species interactions in local communities? How do dispersal traits differ among species in a regional pool and are there dispersal guilds? Are there trade-offs among species between dispersal capacity and capacity to endure species interactions? Answers to such questions may lead us to a more robust, process-based understanding of river-floodplain fish metacommunities.
